# Emergency and Prophylactic Extracorporeal Membrane Oxygenation for Patients Undergoing Valve-in-Valve Transcatheter Aortic Valve Implantation With Small Surgical Bioprosthesis: A Report of Four Cases

**DOI:** 10.7759/cureus.66964

**Published:** 2024-08-15

**Authors:** Eri Watanabe, Satoshi Kometani, Joshi Tsutsumi, Tomohide Takei, Mimiko Tabata

**Affiliations:** 1 Anesthesiology, Yamato Seiwa Hospital, Yamato, JPN; 2 Cardiology, Yamato Seiwa Hospital, Yamato, JPN; 3 Anesthesiology, Yokohama City University Medical Center, Yokohama, JPN; 4 Cardiovascular Surgery, Yamato Seiwa Hospital, Yamato, JPN

**Keywords:** veno-arterial extracorporeal membranous oxygen, small surgical bioprosthesis, structural valve degeneration, aortic bioprosthesis, valve-in-valve procedure

## Abstract

Mechanical circulatory support (MCS) using veno-arterial extracorporeal membrane oxygenation (VA-ECMO) is widely implemented as a rescue device in transcatheter aortic valve implantation (TAVI). Although prophylactic VA-ECMO (pECMO) in TAVI is preferable to emergency VA-ECMO (eECMO) in terms of overall survival, there is currently no consensus on the introduction criteria for pECMO. Here, we report four cases of eECMO and pECMO performed in valve-in-valve TAVI (ViV-TAVI) with a small surgical bioprosthesis to consider the validity of the current pECMO indications. In the two cases that were placed on eECMO, a 19 mm and 21 mm Carpentier-Edwards perimount bioprosthesis (CEP), a stented bioprosthetic valve, were sewn. After the transcatheter heart valve (THV) passed through the surgical aortic valve, acute aortic regurgitation developed, thus leading to hemodynamic instability requiring cardiopulmonary resuscitation, and therefore VA-ECMO was introduced. In the two cases using pECMO, 19 mm of CEP were sewn, and the THV was safely placed once MCS had been established. In all four cases, the patients were removed from VA-ECMO in the operating room following ViV-TAVI. The eECMO patients were discharged on postoperative days 12 and 20, while the pECMO patients were discharged on postoperative days 7 and 9, respectively.

From our experience and based on the findings of some published reviews, ViV-TAVI with a small surgical bioprosthesis is considered to belong to a high-risk patient group demonstrating hemodynamic instability. The introduction of pECMO for such cases may therefore be an effective option for obtaining a better prognosis.

## Introduction

With the spread and development of transcatheter aortic valve implantation (TAVI), mechanical circulatory support (MCS) using veno-arterial extracorporeal membrane oxygenation (VA ECMO) has been widely implemented in the perioperative period. A literature review reported that the use of VA-ECMO in TAVI was 1.9% [1]. Veno-arterial extracorporeal membrane oxygenation has been particularly used in emergency cases for life-threatening TAVI-related periprocedural complications such as cardiac tamponade, severe paravalvular regurgitation, cardiogenic shock, aortic valve annulus rupture, ventricular perforation, ventricular arrhythmias, coronary obstruction, prosthesis migration, and persistent impaired contractility after TAVI deployment. A severely reduced left ventricular ejection fraction may increase the risk of developing hemodynamic instability during the procedure [2]. Prophylactic implantation of VA-ECMO has also been used to achieve hemodynamic stabilization or cardiac unloading in high-risk TAVI cases, such as a severely impaired left ventricular function, a slow recovery from rapid pacing or a high vasopressor requirement during the induction of anesthesia [3]. The survival rate for prophylactic VA-ECMO (pECMO) in TAVI is preferable to that of emergency VA-ECMO (eECMO) because pECMO is 100% whereas eECMO is 61% [1]. There is currently no consensus on the criteria for the introduction of pECMO. We report four cases of eECMO and pECMO performed in valve-in-valve TAVI (ViV-TAVI) with a small surgical bioprosthesis that appeared to have acute aortic regurgitation (AR) to consider the validity of pECMO indication. Written informed consent was obtained from all patients for publication of this report. This study adheres to the CAse REports (CARE) guidelines.

## Case presentation

Emergency VA-ECMO (case 1)

The patient was an 83-year-old female. The Society of Thoracic Surgeons Predicted Risk of Mortality (STS-PROM) score was 6.9%, and the Canadian Study of Health and Aging (CSHA) clinical frailty scale was 4. The patient had a surgical aortic valve replacement (SAVR) with a Carpentier-Edwards perimount bioprosthesis (CEP) 19 mm (Edwards Lifesciences, Irvine, CA, USA) 15 years ago. The patient was observed to have structural valve degeneration (SVD) with preserved left ventricular ejection fraction (LVEF). Intraoperative transthoracic echocardiography (TTE) showed trivial AR and ViV-TAVI was implemented.

**Figure 1:**
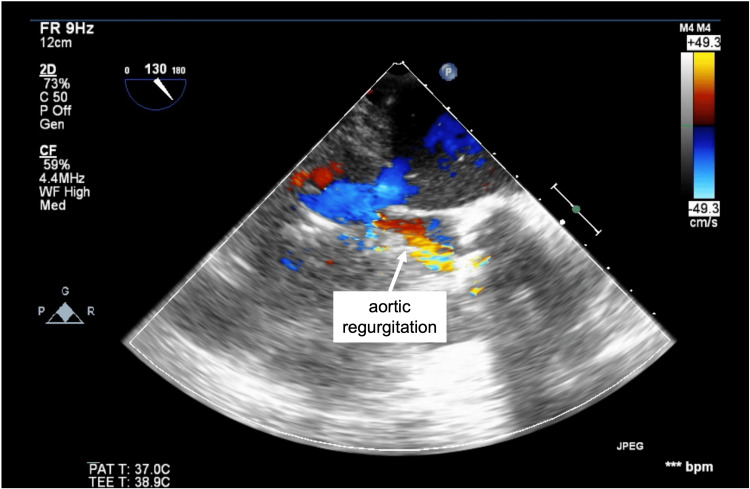
Acute moderate aortic regurgitation observed after transesophageal echocardiography insertion (case 1)

For the transcatheter heart valve (THV), Evolut Pro+ 23 mm (Medtronic, Minneapolis, MN, USA), which is a supraannular self-expanding valve (SEV), was used to ensure a larger effective orifice area (EOA). The procedure was initiated via a transfemoral approach under monitored local anesthetic care. When the THV delivery system was passed through the aortic valve, the diastolic blood pressure decreased significantly, and it was determined that acute AR had occurred due to the restriction of valve movement. Subsequently, THV placement was attempted under controlled pacing, but blood pressure decreased. The patient went into shock and did not respond to catecholamine administration, which led to ventricular fibrillation. Intratracheal intubation was conducted, and general anesthesia was administered. Transesophageal echocardiography (TEE) was inserted, and acute moderate AR was observed (Figure 1). Cardiopulmonary resuscitation (CPR) was performed immediately, after which eECMO was introduced from the femoral artery and vein 9 minutes later. The hemodynamic status following the establishment of MCS was stable with no life-threatening arrhythmias, and the surgery was completed by withdrawing VA-ECMO after THV placement. The patient was removed from the ventilator on postoperative day 1 and left the ICU on postoperative day 2. The patient was discharged from the hospital on postoperative day 12 without complications.

Emergency VA-ECMO (case 2)

The patient was an 83-year-old male with a Society of Thoracic Surgeons Predicted Risk of Mortality (STS-PROM) score of 5.6%, and a CSHA Clinical Frailty Scale score of 4. A SAVR with a 21 mm CEP (Edwards Lifesciences) was performed 15 years previously. The patient was observed to have SVD with preserved LVEF and ViV-TAVI was implemented.

For THV, Evolut FX 23 mm (Medtronic) was used. The procedure was performed using a transfemoral approach after administering general anesthesia. Acute moderate AR due to the restriction of valve movement appeared when the THV delivery system passed through the aortic valve. Subsequently, THV placement was attempted under controlled pacing, but the blood pressure decreased, and low output syndrome was observed. The delivery catheter system was moved up to the ascending aorta, and extracorporeal eECMO was selected as the course of treatment. However, ventricular fibrillation developed during circuit preparation, so CPR was performed. Veno-arterial extracorporeal membrane oxygenation was introduced from the femoral artery and vein 6 minutes later. The hemodynamic status after the establishment of MCS was stable with no life-threatening arrhythmias, and the surgery was completed by withdrawing VA ECMO after THV placement. The patient was removed from the ventilator on postoperative day 1 and left the ICU on postoperative day 3. The patient was discharged from the hospital on postoperative day 20 without complications.

Prophylactic VA-ECMO (cases 3 and 4)

An 84- and 87-year-old female with STS-PROM scores of 9.5% and 10.2% and CSHA clinical frailty scale scores of 4 and 3, respectively were observed to have SVD with preserved LVEF after SAVR with a CEP of 19 mm (Edwards Lifesciences). Therefore, ViV-TAVI was performed.

For THV, Evolut FX 23 mm (Medtronic) was used in both cases. Based on the experience of the above two cases that implemented eECMO, we considered ViV-TAVI for small surgical bioprostheses (label size ≤ 21 mm) as a high-risk group for hemodynamic instability and adapted pECMO. The procedure was performed using a transfemoral approach, and VA-ECMO was introduced from the femoral artery and vein after general anesthesia was administered. Mild acute AR was observed after the THV delivery system passed through the aortic valve.

**Figure 2:**
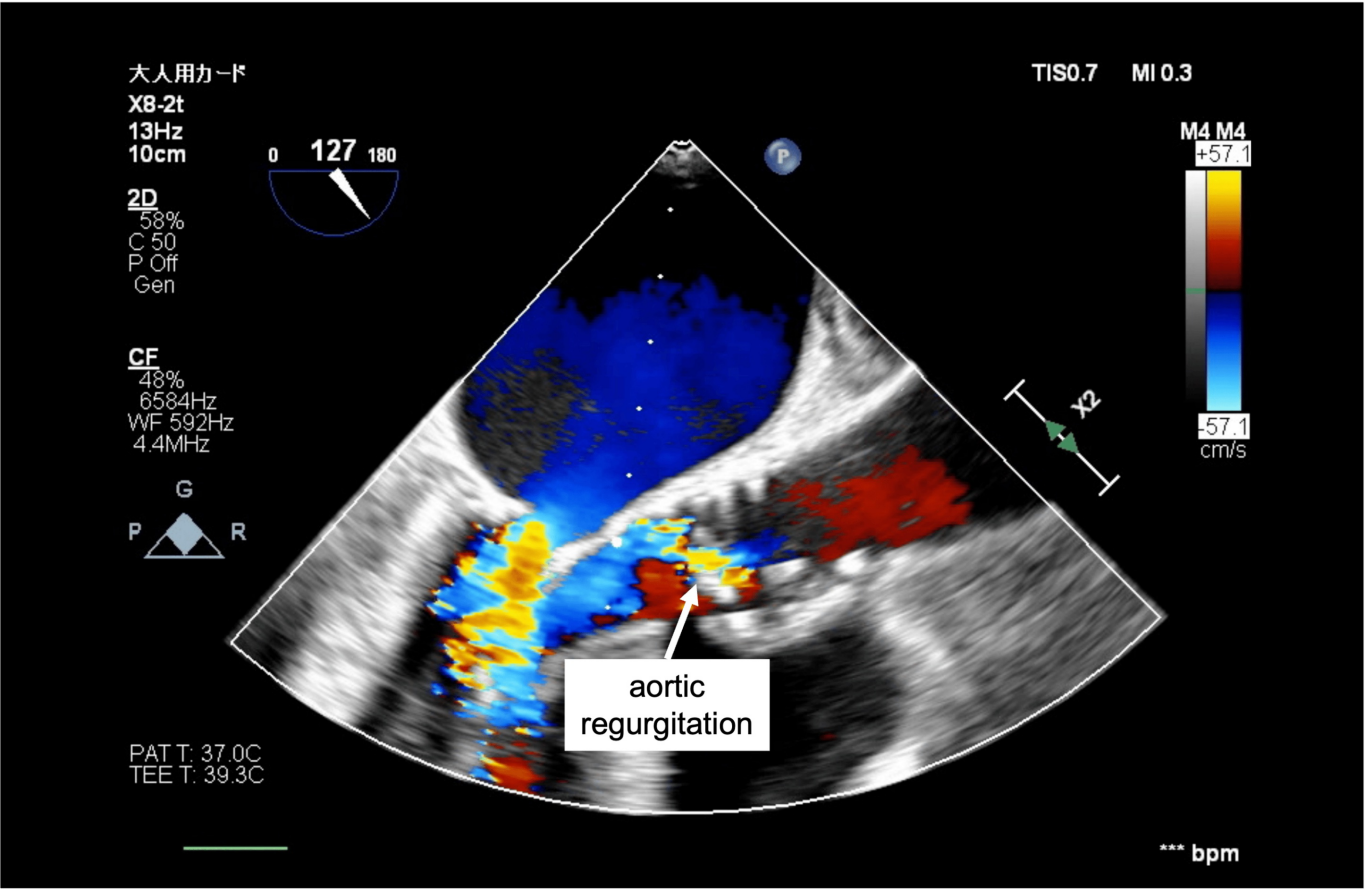
Increased aortic regurgitation observed during the transcatheter heart valve placement (case 3)

**Figure 3:**
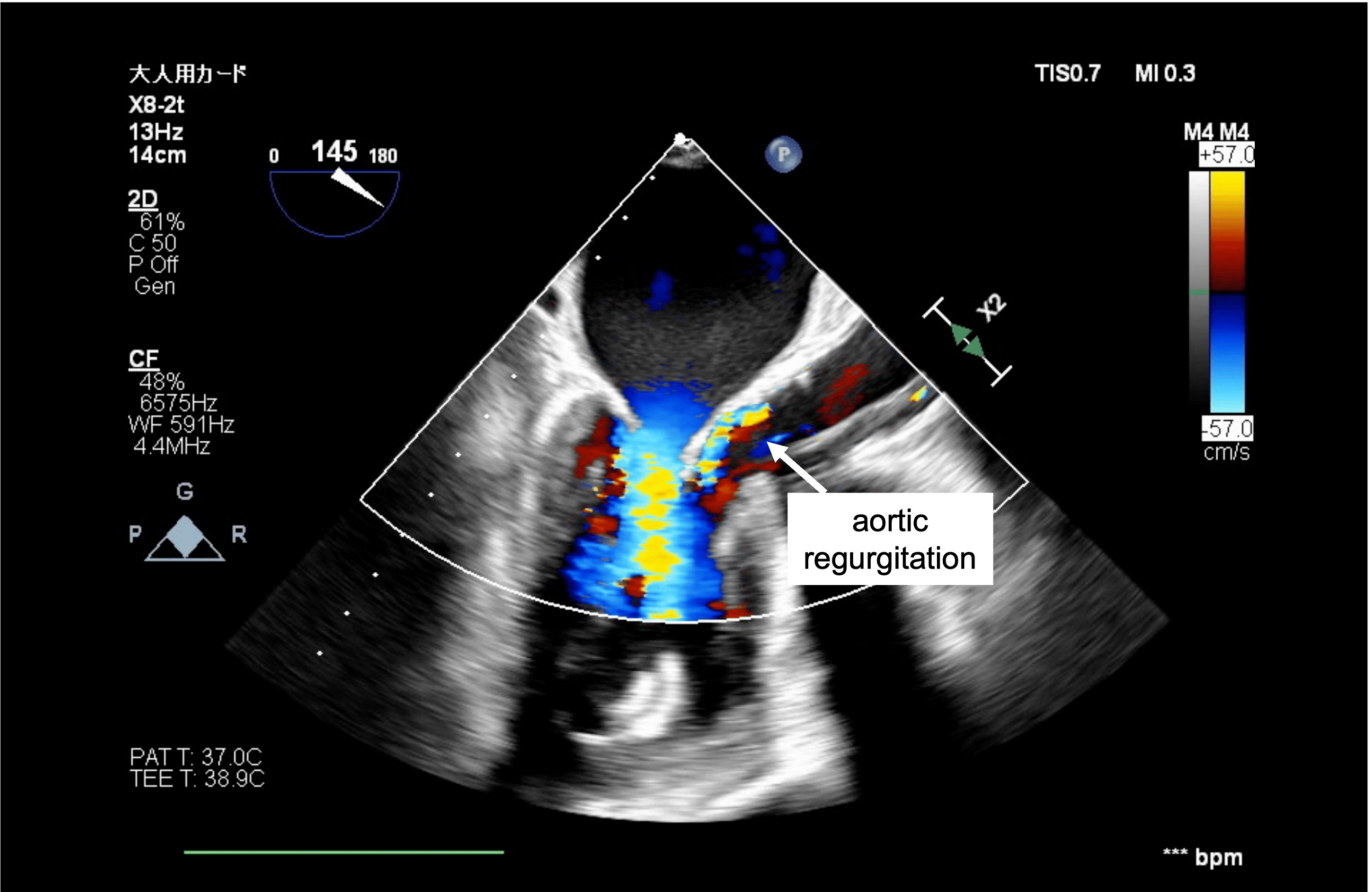
Increased aortic regurgitation observed after the transcatheter heart valve placement (case 4)

In case 3, AR worsened during the THV placement (Figure 2) and in case 4, AR worsened after the THV placement (Figure 3). The ECMO flow rate was increased and both cases were free from any life-threatening arrhythmias under MCS. Surgery was completed by withdrawing VA-ECMO after THV placement under controlled pacing. After extubation, both patients were safely brought to the ICU. The patients left the ICU on postoperative day 3 and were discharged on postoperative days 7 and 9. Table 1 summarises the clinical, echocardiographic, and perioperative characteristics in all four cases.

**Table 1:** Patient characteristics BSA: Body surface area, STS-PROM: The Society of Thoracic Surgeons Predicted Risk of Mortality, CSHA: The Canadian Study of Health and Aging, CEP: Carpentier-Edwards perimount bioprosthesis, ECMO: Extracorporeal membrane oxygenation

	Emergency ECMO		Prophylactic ECMO	
Case	1	2	3	4
Clinical characteristics				
Age (years)/sex	83/F	83/M	84/F	87/F
BMI, kg/m^2^	24.1	26.8	20.5	22.2
BSA, m^2^	1.44	1.68	1.31	1.48
STS-PROM score, %	6.9	5.6	9.5	10.2
CSHA clinical frailty scale	4	4	4	3
Surgical valve prosthesis size, mm	CEP 19mm	CEP 21mm	CEP 19mm	CEP 19mm
Echocardiographic characteristics				
Left ventricular ejection fraction, %	60	69	61	75
Maximal aortic flow velocity, m/sec	5.0	4.3	4.2	4.1
Effective orifice area, cm^2^	0.62	0.65	0.93	0.81
Aortic regurgitation	trivial	trivial	None	trivial
Perioperative characteristics				
Transcatheter heart valve	Evolut pro+ 23mm	Evolut FX 23mm	Evolut FX 23mm	Evolut FX 23mm
Operation time, minutes	205	135	115	100
ECMO duration, minutes	38	35	47	22
ICU stay, days	2	3	3	3
Hospital stay, days	12	20	7	9

## Discussion

The ViV-TAVI for post-SAVR SVD has been reported to have significantly lower postoperative short-term mortality than redo SAVR [4]. Therefore, it is a good option for patients at high risk. In Japan, transcatheter aortic valves (TAV) in surgical aortic valves (SAV) became eligible for insurance coverage in 2018, and TAV-in-TAV in 2023. Therefore, the number of cases is expected to increase.

Regarding long-term prognosis, the one-year mortality was significantly greater with an elevated (≥ 20 mmHg) post-procedural mean gradient [5]. Similarly, patients with small surgical valves (label size ≤ 21 mm) [6] and pre-existing severe prosthesis-patient mismatch (indexed EOA < 0.65 cm2/m2 if the body mass index is < 30 kg/m2 and < 0.6 cm2/m2 if the body mass index is ≥ 30 kg/m2) [7] are independently associated with increased risk for one-year mortality following the ViV procedure. Based on the above, ViV-TAVI with a small surgical bioprosthesis requires ensuring a lower post-procedural mean gradient and larger EOA; consequently, SEV, which is a supra-annular valve, is said to work better than a balloon-expanding valve (BEV) in this regard [8,9]. Surgical ring fracture using a high-pressure balloon is considered effective in reducing the postprocedural mean gradient and increasing the EOA of THV, which are performed especially when using BEV [10]. However, it is for off-label use, and thus not performed in Japan. Furthermore, SEV tends to be preferred because the insurance application for TAV-in-SAV in Japan has been approved for the Evolut series in advance. The SEV was also selected for all cases in this study.

The short-term prognosis of TAVI is mainly related to life-threatening procedural complications, with MCS proposed as a prophylactic or emergency strategy for these difficult situations. To achieve hemodynamic stability during the procedure, pECMO is introduced primarily in cases at high risk for developing hemodynamic instability during and after the THV placement, such as in those cases with poor cardiac function. In previous series and reviews [1,3], the survival rates were 100%. On the other hand, many of the eECMO procedures were used for cases in which structural damage occurred, such as coronary occlusion, aortic valve annulus rupture, and valve position abnormalities. The survival rates of these patients were much lower, at approximately 60%. Thus, although the context of pECMO and eECMO are not the same, ECMO should be introduced without delay by assessing the high risk of hemodynamic instability and complications for TAVI beforehand.

In this report, all four cases were successfully withdrawn from ECMO in the operating room regardless of whether they used pECMO or eECMO. Therefore, the difference in short-term prognosis was not clear. However, eECMO required a longer surgical time, mechanical ventilation time, and hospitalization period. Since the effect of chest compression in the resuscitation of patients with AS becomes limited due to the narrowing of the aortic valve orifice area [11], we believe that pECMO was appropriate in this regard. Currently, there is no consensus on the criteria for the introduction of pECMO in TAVI. Raffa et al. exemplified the high-risk group of hemodynamic instability in TAVI and proposed VA-ECMO using adaptable algorithms [11]. However, there has been no mention of ViV-TAVI or an implanted small surgical bioprosthesis. It is necessary to discuss this by considering the operational status and learning curve of each facility.

The patients in all four cases in this report had preserved cardiac contractile function and were not evaluated as high-risk for TAVI based on the preoperative STS score and clinical frailty scale; therefore, pECMO was not considered initially. We believed that ViV-TAVI with a small surgical bioprosthesis led to hemodynamic instability requiring VA-ECMO due to the rapid increase in left ventricular end-diastolic pressure (LVEDP) associated with the onset of acute AR during the procedure, as well as acute left ventricular failure caused by the delay in subsequent cardiac unloading.

Degenerated surgical bioprostheses exhibit microstructural deformation accompanied by collagen structure destruction and elastin damage through noncalcific mechanisms such as inflammation, oxidative stress, and mechanical stress, in addition to valve calcification, which is also found in autologous valves [12]. As a result, the structure of such degenerated surgical bioprostheses may cause significant acute AR owing to a leaflet with lower flexibility and a limited function when the THV delivery system passes through the aortic valve. As acute AR also occurred in our cases, early cardiac unloading is necessary for situations in which the LVEDP rises rapidly. However, SEV is expected to require more time for device positioning and deployment than balloon-expandable valves. Furthermore, when the SEV contacts annularly, the aortic valve structure is temporarily occluded by the deploying valve, resulting in a further increase in LVEDP. To achieve cardiac unloading, the operator must then rotate the deployment knob and deploy the device until the supra-annular valve functions and the blood pressure is restored. However, structural and hemodynamic changes from aortic occlusion to unloading due to this annular contact may be prolonged by earlier annular contact for small surgical bioprostheses. Based on this experience, we determined that ViV-TAVI with a small surgical bioprosthesis belongs to the high-risk group with a combination of factors that increase LVEDP. In particular, hemodynamic breakdown should be considered in supra-annular SEVs, which require time for device positioning and deployment.

## Conclusions

We determined that patients requiring ViV-TAVI with a small surgical bioprosthesis are a high-risk group for intraoperative hemodynamic instability based on our experience with four cases that received eECMO and pECMO. Currently, there is no consensus on the introduction criteria for pECMO in high-risk TAVI. However, in ViV-TAVI with a small surgical bioprosthesis, MCS by pECMO for organ oxygenation and securing coronary artery blood flow, is a reasonable and safe procedure.
